# RBM25 Drives Hepatocellular Carcinoma Progression by Stabilizing YAP Through Regulating Oncogenic Splicing‐switch of MYPT1

**DOI:** 10.1002/advs.77792

**Published:** 2026-09-16

**Authors:** Wenjing Zhang, Lili Zhi, Tian Huang, Lingya Feng, Chaoqun Chen, Huanhuan Wei, Xiaolong Liu, Lei Chen, Jinrui Zhang, Ge Zhang, Baofeng Zhao, Rong Liu, Dan Chen, Yangfan Qi, Yang Wang

**Affiliations:** ^1^ Sino‐US Research Center for Cancer Translational Medicine of the Second Affiliated Hospital of Dalian Medical University & Institute of Cancer Stem Cell Dalian Medical University Dalian China; ^2^ Sir Run Run Shaw Hospital Zhejiang University School of Medicine Hangzhou China; ^3^ CAS Key Laboratory of Computational Biology Bio‐Med Big Data Center Shanghai Institute of Nutrition and Health University of Chinese Academy of Sciences Chinese Academy of Sciences Shanghai China; ^4^ Department of Immunology College of Basic Medical Sciences Dalian Medical University Dalian China; ^5^ State Key Laboratory of Medical Proteomics, National Chromatographic R. & A. Center Dalian Institute of Chemical Physics Chinese Academy of Sciences Dalian China; ^6^ Translational Cancer Research Center Peking University First Hospital Beijing China; ^7^ Department of Pathology First Affiliated Hospital Dalian Medical University Dalian China; ^8^ Soochow University Cancer Institute Suzhou China

**Keywords:** alternative splicing, HCC, MYPT1, RBM25, YAP

## Abstract

Deregulated alternative splicing (AS) is a hallmark of hepatocellular carcinoma (HCC), yet the specific splicing factors driving oncogenic programs remain largely uncharacterized. Here, we identify RNA‐binding motif protein 25 (RBM25) as a potent oncogenic driver that is overexpressed in HCC and correlates with dismal patient prognosis. Functionally, RBM25 depletion impairs HCC progression across in vitro models, in vivo xenografts, and patient‐derived organoids. Integrated transcriptomic and interactome profiling reveals that RBM25 orchestrates a specific AS landscape, most notably promoting exon 13 inclusion of MYPT1. Mechanistically, RBM25 recruits PRPF40A to facilitate the production of the oncogenic MYPT1‐L isoform. This isoform switch acts as a molecular stabilizer for the transcriptional co‐activator YAP, thereby sustaining Hippo pathway dysregulation and tumor growth. Finally, a high‐throughput screen of the US drug collection identified candicidin as a small‐molecule inhibitor that suppresses RBM25 expression, effectively phenocopying RBM25 knockdown. Our findings define the RBM25‐MYPT1‐YAP axis as a critical vulnerability in HCC and nominate RBM25 as a viable prognostic biomarker and therapeutic target.

## Graphical abstract

This study establishes that the RBM25‐PRPF40A interaction modulates MYPT1 splicing, promoting the production of the oncogenic long isoform. This isoform stabilizes YAP by suppressing its phosphorylation and subsequent proteasomal degradation, ultimately accelerating tumor growth. Candicidin is identified as a potent RBM25 inhibitor that effectively impairs tumorigenesis in patient‐derived HCC organoids.

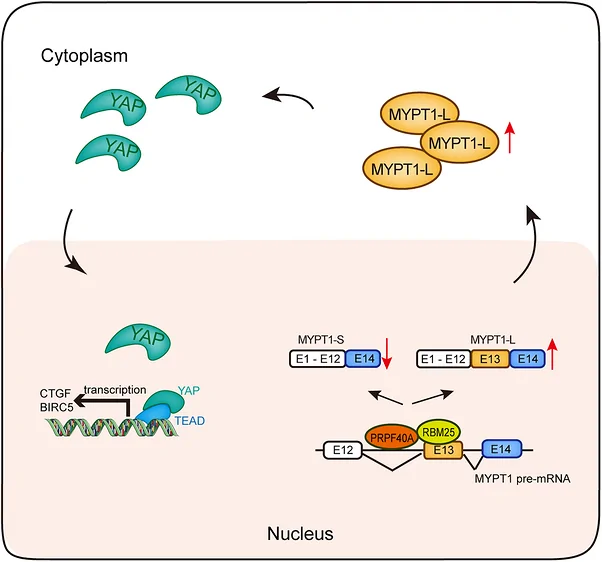

## 1 Introduction

Primary liver cancer is the sixth most commonly diagnosed cancer and the third leading cause of cancer death worldwide, accounting for 7.8% of all cancer deaths [1]. Hepatocellular carcinoma (HCC) is the most prevalent type of primary liver cancer, constituting 75–85% of all cases [1]. The main risk factors for HCC include chronic infection with hepatitis B or hepatitis C, alcohol addiction, metabolic liver disease, and aflatoxin‐contaminated foods [2]. Current therapeutic approaches for early and mid‐stage of HCC encompass surgical resection, liver transplantation, local ablation and arterial chemoembolization [3]. However, the majority of HCC cases are diagnosed at an advanced stage when curative treatment options are limited, leading to a reduced 5‐year survival rate. Therefore, new potential biomarkers and therapeutic targets are urgently needed to improve the survival of HCC patients.

Alternative splicing (AS) of pre‐mRNA is a crucial post‐transcriptional mechanism in complex organisms that allows for the production of multiple transcript isoforms from a single gene, contributing to proteome diversity. AS events are tightly regulated by numerous factors, and their dysregulation has been observed in various diseases, including cancer [4]. Aberrant splicing can induce the production of noncanonical and cancer‐specific mRNA transcripts, which can be translated into distinct protein isoforms that contribute to hallmarks of cancer progression, such as sustaining proliferative signaling, evading growth suppression, resisting cell death, triggering invasion and metastasis, avoiding immune surveillance, and developing drug‐resistance [5, 6, 7]. Importantly, aberrant RNA alternative splicing is involved in liver cancer development and malignancy [8, 9, 10, 11], but the impact of these variants on liver cancer remains largely unknown. Therefore, the study of alternative splicing regulation in HCC may provide new potential biomarkers and therapeutic targets for liver cancer.

RNA‐binding motif protein 25 (RBM25) is an RNA binding protein, structured with an N‐terminal RRM region, an intermediate RE/RD region, and a C‐terminal PWI region [12]. It's primarily localized in nuclear speckles and functions as a regulator of alternative splicing. RBM25 plays key roles in apoptotic cell death by regulating the apoptotic regulator Bcl‐x and MYC inhibitor BIN1 [12, 13, 14]. Emerging evidence suggests that RBM25 plays a crucial role in cancer‐related alternative splicing, presenting a potential therapeutic target. While bioinformatics analyses have indicated that RBM25 is overexpressed in HCC patients [15], the precise molecular mechanisms by which RBM25 contributes to HCC progression remain largely unexplored.

Yes‐associated protein (YAP) is a pivotal transcriptional regulator involved in key cellular processes such as proliferation, survival, and differentiation [16, 17]. It is widely expressed in human malignancies and plays a vital role in the initiation, progression, and growth of various solid tumors [16, 17]. As a central effector of the Hippo signaling pathway, YAP regulation has been studied through its phosphorylation and subcellular localization [18]. These findings offer critical insights into the fine‐tuning of YAP function during tumorigenesis, highlighting the potential of targeting YAP as a novel therapeutic strategy for cancer treatment.

In this study, we reported that RBM25 is frequently upregulated in HCC patients, and its overexpression is associated with poor prognosis. We conducted RNA‐seq and identified RBM25 regulated alternative splicing events. Further investigation revealed that knockdown of RBM25 inhibited cell proliferation and tumor growth by switching the AS of MYPT1 gene. The suppression of RBM25, as exemplified by candicidin identified in this study, exhibited potent inhibitory effects on HCC cells and organoids. Our findings suggest that RBM25 is a crucial oncogenic splicing factor and could serve as a potential therapeutic target in HCC.

## 2 Results

### 2.1 RBM25 is Increased in HCC and its Overexpression is Associated With Poor Prognosis

**FIGURE 1:**
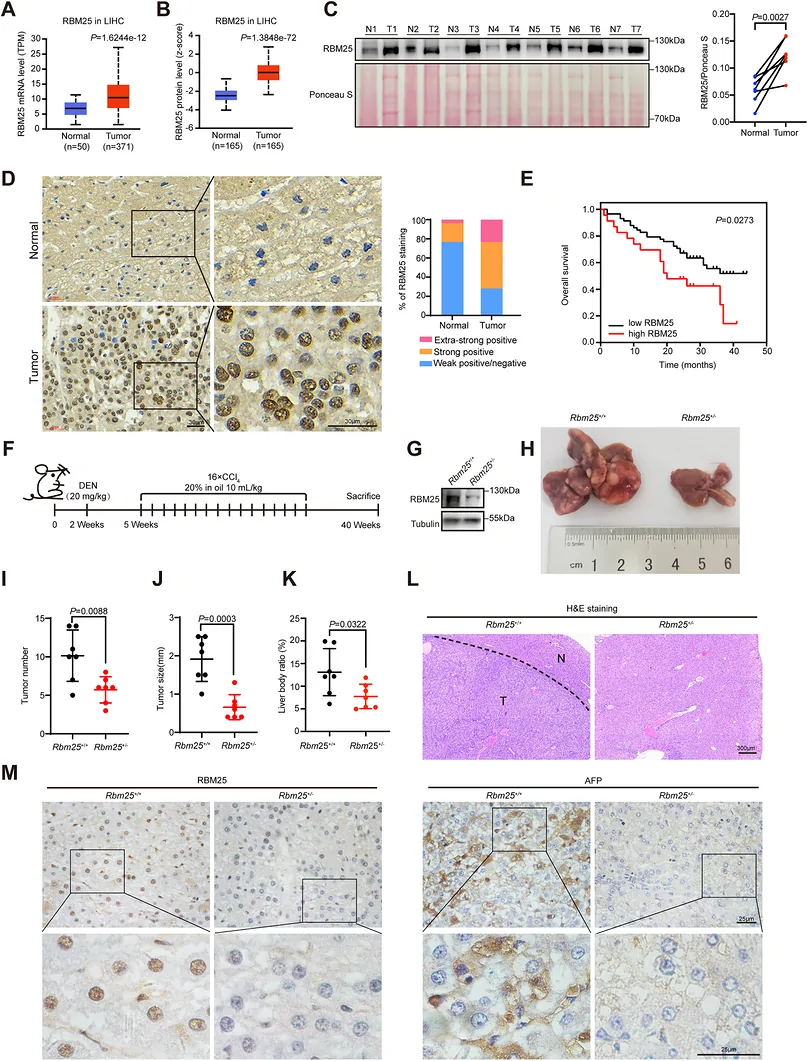
RBM25 is associated with HCC progression and prognosis in human and mice. (A) The mRNA level of RBM25 in HCC samples (n = 371) and normal liver samples (n = 50) from TCGA database via UALCAN portal. (B) The protein level of RBM25 in HCC samples (n = 165) and normal liver samples (n = 165) from CPTAC via UALCAN portal. (C) Protein levels of RBM25 in the paired fresh‐frozen HCC tissues (T) and the adjacent normal tissue specimens (N) from HCC patients were examined by a western blot assay (n = 7). The relative RBM25 protein levels were quantified. (D) Immunohistochemical (IHC) staining of RBM25 in normal liver tissues and HCC tissues (n = 82). Scale bar = 30 µm. The quantification of the percentage of cases exhibited negative, weak positive, strong positive or extra‐strong positive IHC staining of RBM25. (E) Kaplan‐Meier curve showing overall survival of HCC patients with high or low RBM25 expression based on immunohistochemical microarray analysis (n = 82). (F) Scheme used to establish the DEN/CCl_4_ induced HCC mouse model. (G) The protein levels of RBM25 in livers from RBM25 wild type or RBM25 heterozygous deletion mice were measured by a western blot assay. (H) Representative photographs of intrahepatic tumor tissues in the RBM25 wild type and RBM25 heterozygous deletion mice 40 weeks after birth. (I) Numbers of tumor nodules in the indicated mice (n = 7 mice per group). (J) Tumor sizes in the indicated mice (n = 7 mice per group). (K) Liver body ratio in the indicated mice (n = 7 mice per group). (L) H&E staining of intrahepatic tumor tissues in the RBM25 wild type and RBM25 heterozygous deletion mice 40 weeks after birth. Scale bar: 60 µm. (M) Immunohistochemistry was used to examine the expression of RBM25 (left) and AFP (right) in liver tissues of RBM25 wild type and RBM25 heterozygous deletion mice in DEN/CCl_4_ induced HCC mouse model. Scale bar: 25 µm. *P* values were calculated by two‐tailed Student's t test (A‐C, I‐K) or log‐rank (Mantel‐Cox) test (E).

To investigate the involvement of RBM25 in hepatocarcinogenesis, we first examined its expression in HCC tissues. We analyzed RBM25 mRNA expression in primary HCC tissue samples (n = 371) and normal liver tissue samples (n = 50) from TCGA datasets using the UALCAN platform (https://ualcan.path.uab.edu/analysis.html). This analysis revealed a significant enrichment of RBM25 mRNA level in HCC compared to normal tissue (Figure 1A). The protein level of RBM25 in HCC tissues was also elevated compared to normal tissues (Figure 1B). Subsequently, we surgically collected paired liver samples and adjacent normal tissues from seven patients to measure RBM25 levels. Consistently, the RBM25 protein expression levels were significantly increased in HCC specimens compared with paired normal tissues (Figure 1C). Such increase was independently validated by an immunohistochemistry assay of 82 paired HCC tissues and matched adjacent normal tissues (Figure 1D). Specifically, more than 70% of liver cancer samples exhibited strong or extra‐strong staining for RBM25. In contrast, 76% of adjacent normal liver tissues displayed negative or weak staining for RBM25 (Figure 1D). Furthermore, survival analysis indicated that patients with higher expression of RBM25 exhibited a poor prognosis (Figure 1E). In addition, Kaplan‐Meier analyses using TCGA data showed that patients with higher levels of RBM25 had shorter overall survival (OS), recurrence‐free survival (RFS), disease‐specific survival (DSS) and progression free survival (PFS) (Figure S1A). Taken together, these data strongly suggest that upregulation of RBM25 is closely associated with HCC progression, and RBM25 is a potential prognostic biomarker for liver patients.

### 2.2 Loss of RBM25 Inhibits HCC Tumorigenesis In Vivo

To explore the role of RBM25 in hepatocarcinogenesis in vivo, we used diethylnitrosamine and carbon tetrachloride (DEN/CCl_4_) induced HCC models (Figure 1F). We first generated genetically engineered mouse models of Rbm25 using CRISPR/Cas9‐mediated gene editing to delete exon 2 to exon 18 of *Rbm25* (Figure S1B). Homozygous knockout of *Rbm25* (*Rbm25^−/−^* mice) resulted in embryonic lethality, hence we used heterozygous *Rbm25* mice (*Rbm25^+/−^* mice) to assess the impact of RBM25 on hepatocarcinogenesis. The *Rbm25^+/−^* KO mice were confirmed by PCR using specific primers, gene sequencing and western blotting (Figure 1G and Figure S1C,D). We then established an HCC mouse model using DEN/CCL_4_, which involved the injection of 20 mg/kg of DEN, followed by intraperitoneal injection of CCL_4_ for 16 weeks. The tumor burden was analyzed at 40 weeks of age (Figure 1F). Compared with wild type mice, *Rbm25^+/−^* mice exhibited a significant reduction in tumor number, size, and the liver: body weight ratio (Figure 1H‐K). Consistently, we observed smaller histological lesions and reduced alpha‐fetoprotein (AFP) staining in the livers of *Rbm25^+/−^* mice (Figure 1L,M). Collectively, these findings indicate that loss of RBM25 inhibits hepatocarcinogenesis in mice.

### 2.3 RBM25 Promotes Tumorigenic Potentials of HCC Cells

**FIGURE 2:**
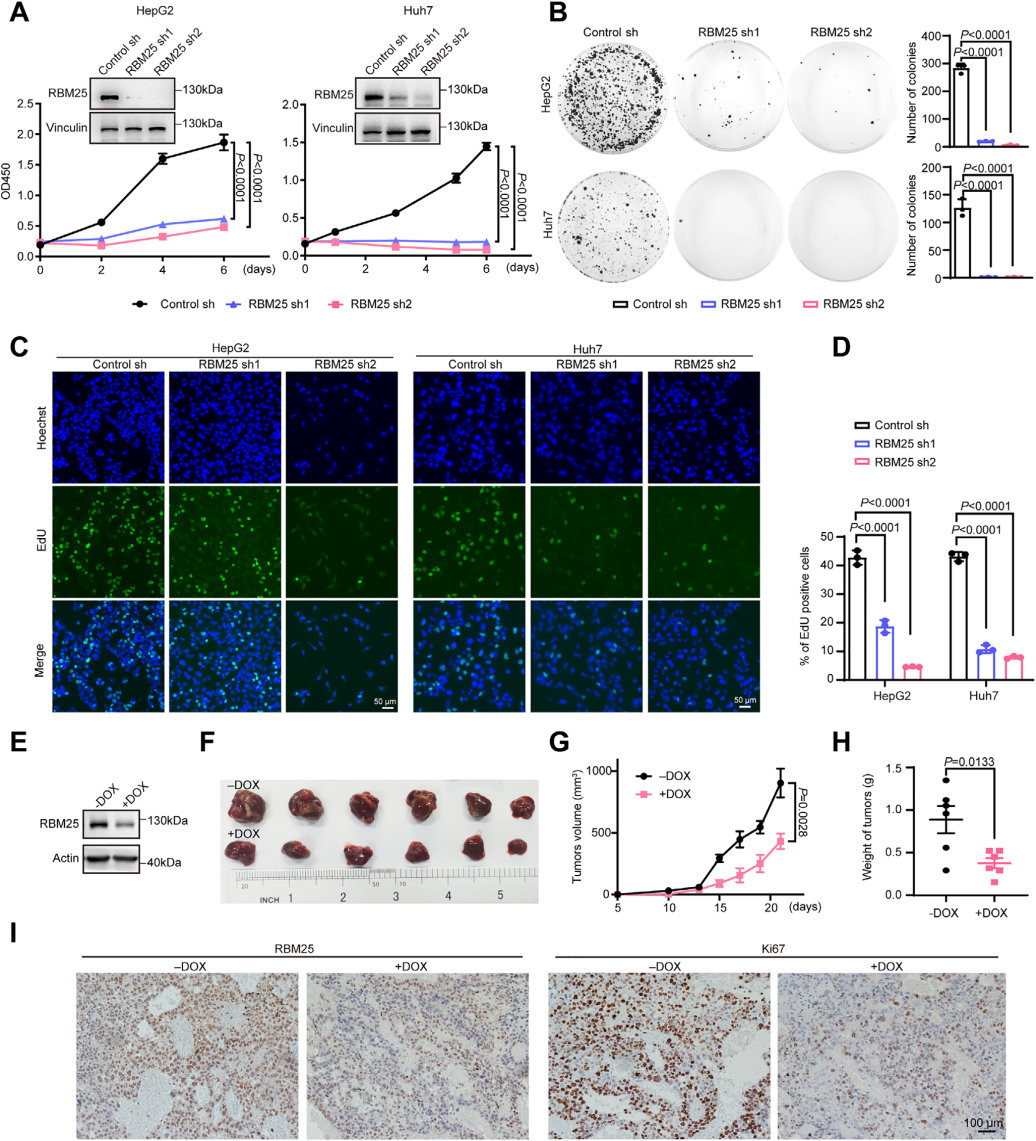
RBM25 ablation inhibits the growth of HCC cells. (A) Cell viabilities of HepG2 and Huh7 cells with RBM25 knockdown were measured by the CCK‐8 growth curve assays (n = 4, mean ± SD). (B) Colony formation assays using HepG2 and Huh7 cells with stable depletion of RBM25 were performed. Three experiments were carried out, with mean ± SD of colony numbers plotted. (C, D) The proliferative abilities of HepG2 and Huh7 cells with stable depletion of RBM25 were detected with an EdU staining assay. Three experiments were carried out, with mean ± SD of colony numbers plotted. (E) The protein levels of RBM25 in Huh7 cells with DOX‐inducible depletion of RBM25 were measured with or without DOX treatment by a western blot assay. (F–I) Xenograft tumors were generated using NYG mice subcutaneously injected with Huh7 cells with DOX‐inducible depletion of RBM25. Mice were fed water with or without DOX. (F) Pictures of the tumors removed after 22 days. (G) The average sizes of xenograft tumors were measured and plotted (n = 6, error bars indicate mean ± SEM). (H) Tumors were weighed and plotted (n = 6, mean ± SEM). (I) Immunohistochemical staining of RBM25 and Ki67 of xenograft tumors. Scale bars: 100 µm. *P* values were determined by 2‐way ANOVA (A, G), 1‐way ANOVA with Dunnett's multiple comparison test (B, D) or unpaired t test (H).

We further investigated the impact of RBM25 knockdown on the tumor growth of HCC cells both in vitro and in vivo. We stably depleted endogenous RBM25 expression using two independent shRNAs. The knockdown of RBM25 was confirmed in four HCC cell lines, including HepG2, Huh7, Hep3B, and HCCLM9 (Figure 2A and Figure S2A). We found that depletion of RBM25 significantly inhibited the growth of these cells, as judged by Cell Counting Kit‐8 (CCK‐8) assays (Figure 2A and Figure S2A). Moreover, RBM25 knockdown notably suppressed anchorage‐dependent growth in HepG2, Huh7, Hep3B, and HCCLM9 cells (Figure 2B and Figure S2B). In line with previous results, RBM25 depletion severely impaired the proliferation in both HepG2 and Huh7 cells, as judged by EdU staining (Figure 2C,D). Conversely, overexpression of RBM25 promoted proliferation in both HepG2 and Hep3B cells (Figure S2C,D). To further determine whether RBM25 reduction inhibits HCC cell growth in vivo, we subcutaneously injected Huh7 cells carrying a doxycycline (DOX)‐inducible shRNA against RBM25 into flanks of NYG mice (n = 6), and then administered water with or without DOX to deplete RBM25 (Figure 2E). We also monitored the body weight of all mice throughout the experiment to evaluate the potential toxic effects induced by RBM25 depletion, and no obvious weight loss was observed between the two groups (Figure S2E). Consistent with our in vitro observations, knockdown of RBM25 significantly suppressed tumor growth and the final tumor weight in vivo (Figure 2F‐H). Meanwhile, Ki67 staining by IHC revealed that depletion of RBM25 inhibited tumor proliferation more strongly than control tumors (Figure 2I). Altogether, our results suggest that loss of RBM25 suppresses liver cancer progression both in cultured cells and in xenograft tumors.

### 2.4 Global Identification of AS Events Regulated by RBM25

**FIGURE 3:**
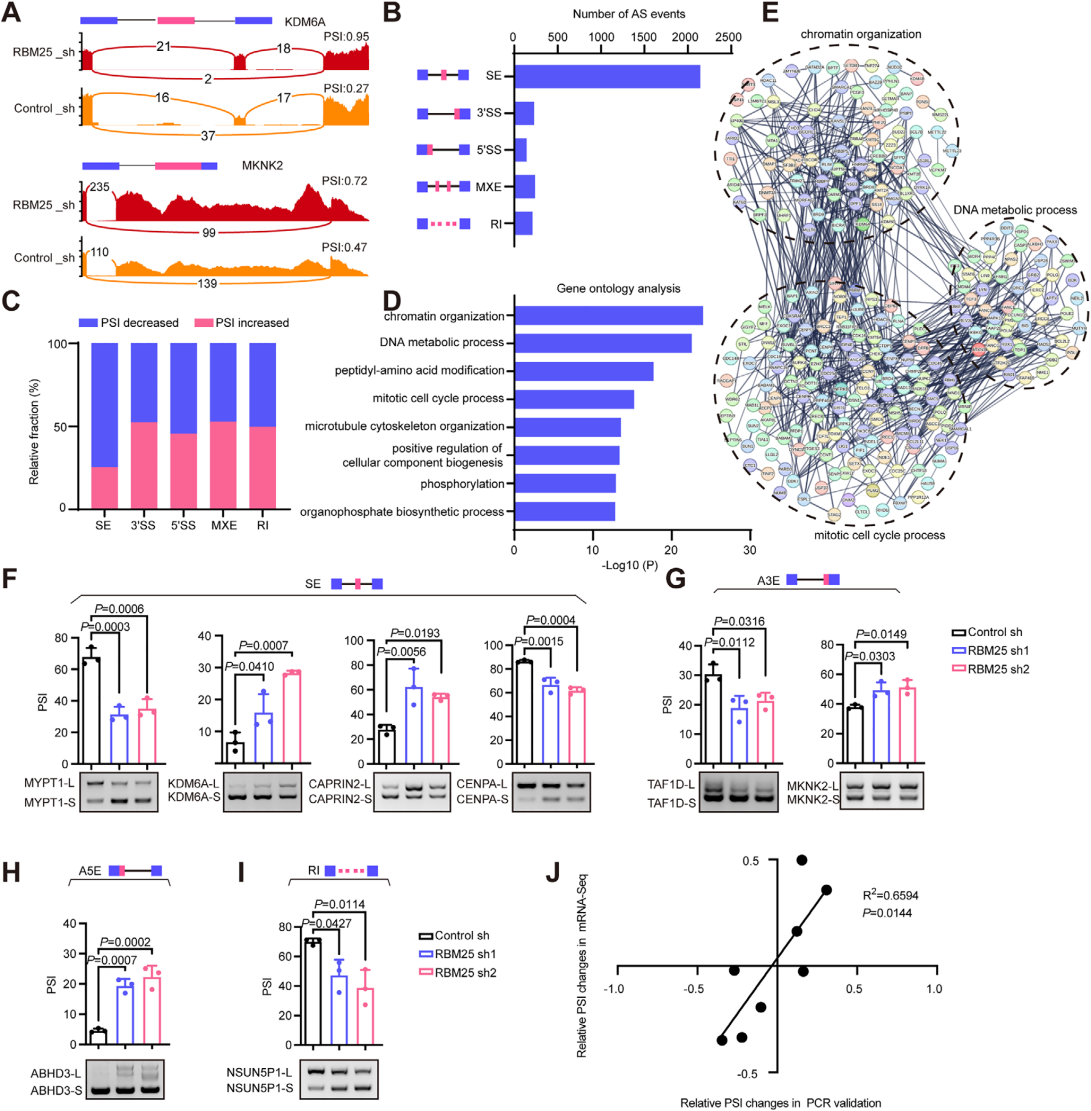
Global identification of AS events regulated by RBM25. (A) Reads coverage plots showing alternative splicing in representative genes (KDM6A and MKNK2) upon RBM25 knockdown. Gene structures are depicted at the top, with the alternative spliced exon highlighted in pink. The curved lines represent splice junction reads (numbered), and PSI values indicate exon inclusion levels. (B) Quantification of the different AS events affected by RBM25. (C) The relative fraction of each AS event positively or negatively affected by RBM25. (D) Gene ontology of RBM25‐regulated AS targets. (E) Functional association network of RBM25‐regulated AS targets using the STRING database, and subgroups are marked according to their functions. (F‐I) Different types of RBM25‐regulated AS events, including SE (F), A3E (G), A5E (H) and RI (I), were verified by semi‐quantitative RT‐PCR using HepG2 cells with stable depletion of RBM25 or control cells. The mean ± SD of PSIs from three experiments were plotted. (J) Correlation between the relative changes in PSI values observed by RNA‐seq vs RT‐PCR confirmation. *P* values were determined by 1‐way ANOVA with Dunnett's multiple comparison test (F‐I).

To gain further insights into RBM25‐regulated AS events involved in hepatocarcinogenesis, we conducted mRNA‐seq with RBM25‐knockdown HepG2 cells. With ∼74 million 150‐nt paired‐end reads, we identified a total of 3119 RBM25‐regulated AS events with an obvious change of percent‐spliced‐in (PSI) values (PSI ≥ 0.15, *P*<0.05) in HepG2 cells. The read tracks of two examples were shown (Figure 3A). RBM25‐regulated AS events could be categorized into 5 AS types, including skipped exon (SE), alternative 3′ ss exon (A3E), alternative 5′ ss exon (A5E), mutually exclusive exons (MXE), and retained intron (RI) (Figure 3B). The majority of these AS events belonged to the skipped exon category. Subsequent analysis suggested that RBM25 acts as a splicing activator, as knockdown of RBM25 induced a decrease in PSI value for the skipped exon events (Figure 3C), aligning with previous report that RBM25 is a global splicing factor promoting the inclusion of alternatively spliced exons [13].

We further analyzed cellular functions of RBM25‐regulated AS events using gene ontology (GO) and found that the splicing targets affected by RBM25 were associated with tumor‐related functions including regulation of chromatin organization, DNA metabolic process, peptidyl‐amino acid modification, mitotic cell cycle process, microtubule cytoskeleton organization, positive regulation of cellular component biogenesis, phosphorylation and organophosphate biosynthetic process (Figure 3D). Many of the RBM25‐regulated splicing targets were functionally connected into well‐linked interaction networks involved in regulation of chromatin organization, DNA metabolic process and mitotic cell cycle process, as determined by STRING (Search Tool for the Retrieval of Interacting Genes/Proteins) (Figure 3E). To verify the accuracy of the RNA‐seq results on AS, we subsequently validated ten RBM25‐affected AS events, which were selected from the most significantly enriched terms associated with the mitotic cell cycle process, DNA metabolic process, and chromatin organization. We confirmed that RBM25 either positively or negatively controls all endogenous AS events tested, including SE, A3E, A5E and RI splicing types (Figure 3F‐I). The relative changes of PSIs obtained from RT‐PCR are highly correlated to those observed by RNA‐seq (Figure 3J).

### 2.5 Depletion of RBM25 Inhibits Liver Cancer Progression Partially Through MYPT1

**FIGURE 4:**
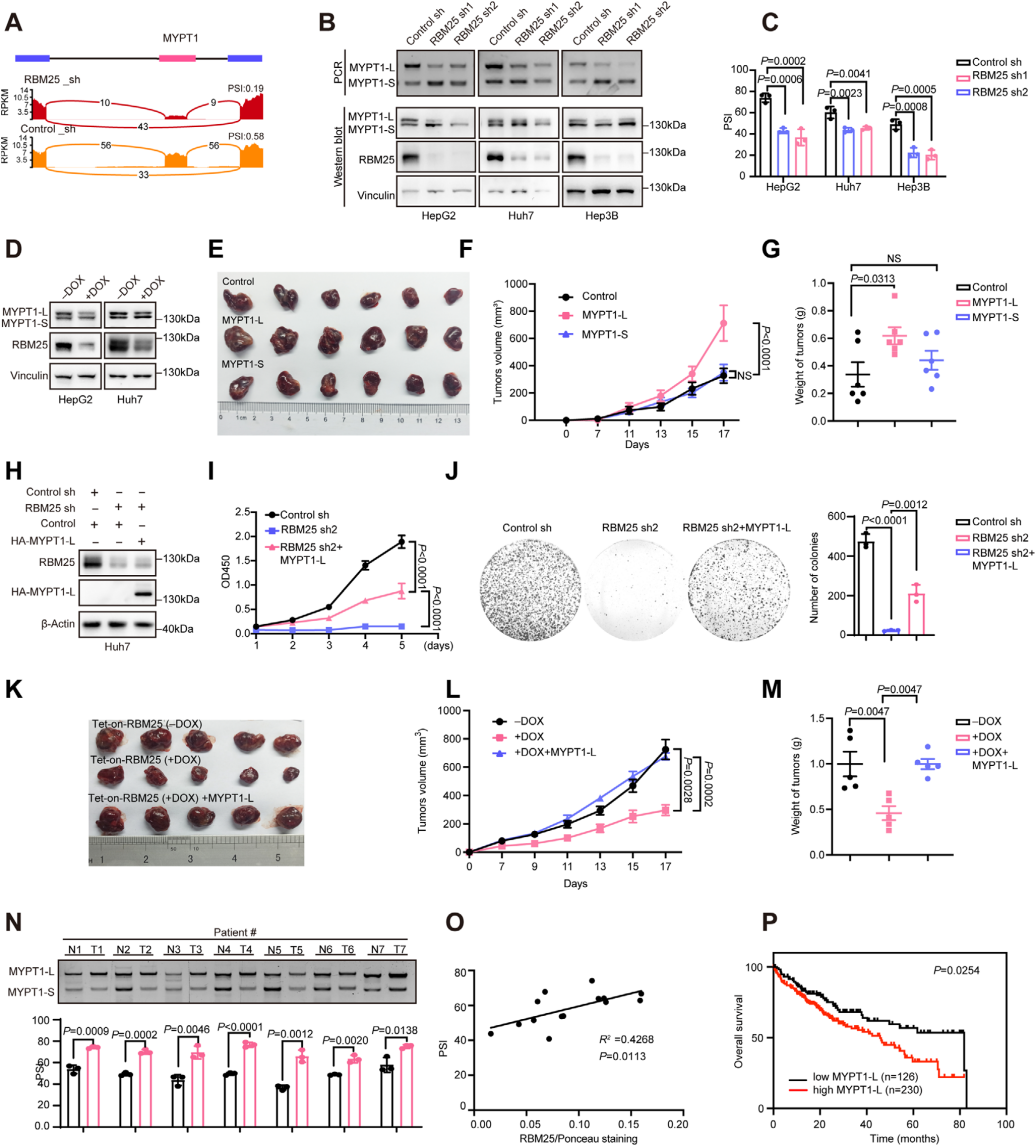
Depletion of RBM25 inhibits liver cancer progression partially through MYPT1. (A) Reads coverage plots of MYPT1 alternative splicing upon RBM25 knockdown. Gene structures are depicted at the top, with the alternative spliced exon highlighted in pink. The curved lines represent splice junction reads (numbered), and PSI values indicate exon inclusion levels. (B, C) The splicing change of MYPT1 was detected by semi‐quantitative RT‐PCR and western blotting in HepG2, Huh7 and Hep3B cells with stable depletion of RBM25 or control cells. The mean ± SD of PSIs from three experiments were plotted. (D) Western blot analysis of MYPT1 splice variants in HepG2, Huh7 cells with DOX‐inducible depletion of RBM25. (E‐G) Xenograft tumors were generated using NYG mice subcutaneously injected with Huh7 cells with expression of MYPT1‐L or MYPT1‐S. (E) Pictures of the tumors removed after 17 days. Scale bar, 1 cm. (F) The average sizes of xenograft tumors were measured and plotted (n = 6, error bars indicate mean ± SEM). (G) Tumors were weighed and plotted. (H) The protein levels of RBM25, HA‐MYPT1‐L and Tubulin were examined by a western blot assay. (I) Cell proliferation of RBM25‐depleted Huh7 cells with or without MYPT1‐L re‐expression was measured by CCK‐8 (n = 5, error bars indicate mean ± SD). (J) Colony formation assays using RBM25‐depleted Huh7 cells with or without MYPT1‐L re‐expression were performed. Three experiments were carried out, with mean ± SD of colony numbers plotted. (K‐M) Xenograft tumors were generated using NYG mice subcutaneously injected with inducible RBM25‐depleted Huh7 cells with or without MYPT1‐L re‐expression. Mice were fed water with or without DOX. (K) Pictures of removed tumors. Scale bar, 1 inch. (L) The average sizes of xenograft tumors were measured (n = 5, mean ± SEM). (M) Tumors were weighed and plotted (n = 5, mean ± SEM). (N) Splicing of MYPT1 in paired HCC tumors and adjacent normal tissues was analyzed by RT‐PCR. PSI values were calculated (n = 3, mean ± SD). (O) Correlation of RBM25 levels in Figure 1B with the PSI values of MYPT1 was analyzed. (P) Kaplan‐Meier analysis of the overall survival of HCC patients with high or low MYPT1‐L expression based on TCGA RNA‐seq data. *P* values were determined by 1‐way ANOVA with Dunnett's multiple comparison test (C, G), 2‐way ANOVA (F, I, L), 1‐way ANOVA with Tukey's multiple comparison test (J, M), two‐tailed Student's t test (N), Pearson correlation test (O) or log‐rank (Mantel‐Cox) test (P).

To elucidate the mechanism by which RBM25 influences cancer progression, we focused on the myosin light chain phosphatase target subunit 1 (MYPT1) gene among the validated RBM25‐affected AS events. This is because its transcripts exhibited a significant shift from exon‐included long isoform (MYPT1‐L) to the exon‐skipped short isoform (MYPT1‐S) in the RNA‐seq of HepG2 cells with RBM25 knockdown (Figure 4A). MYPT1 is a crucial component of the myosin light chain phosphatase (MLCP) protein complex [19]. The splicing shift of MYPT1 resulted in a rapid and substantial decrease of MYPT1‐L upon RBM25 knockdown in HepG2, Huh7, and Hep3B cells as judged by RT‐PCR and western blotting (Figure 4B,C). In addition, an inducible knockdown of RBM25 also switched splicing of MYPT1 in both HepG2 and Huh7 cells (Figure 4D). To further validate RBM25‐mediated splicing regulation, we restored RBM25 expression in RBM25‐depleted HepG2, Huh7 and Hep3B cells. The abnormal MYPT1 splicing pattern caused by RBM25 deficiency was reversed after RBM25 reintroduction (Figure S3A,B).

To investigate whether the RBM25‐mediated splicing regulation of MYPT1 plays the oncogenic role in liver cancer progression, we examined the individual functions of two MYPT1 isoforms separately (MYPT1‐L and ‐S). We stably overexpressed MYPT1‐L or MYPT1‐S in Hep3B and Huh7 cells respectively (Figure S3C). Overexpression of MYPT1‐L significantly promoted the growth of these cells as judged by CCK‐8 and colony formation assays, whereas MYPT1‐S had no obvious effect on cell growth (Figure S3C,D). Furthermore, we designed shRNAs targeting the exon 13 of MYPT1 to specifically deplete the MYPT1‐L isoform in Hep3B and Huh7 cells (Figure S3E). As expected, knockdown of MYPT1‐L notably suppressed the growth of Hep3B and Huh7 cells as determined by CCK‐8 and colony formation assays (Figure S3E,F). To further determine the roles of distinct MYPT1 isoforms in vivo, we established xenograft mouse model with Huh7 cells expressing MYPT1‐L or MYPT1‐S or control vector (n = 6). Tumor volumes were measured every 2 days until the point of animal euthanasia and subsequent tumor extraction. The body weight of mice was monitored and no remarkable weight disparity was identified among three experimental groups (Figure S3G). In line with our in vitro findings, xenografts from the group overexpressing MYPT1‐L exhibited accelerated growth rates and a greater final tumor mass compared to both the control and MYPT1‐S groups (Figure 4E‐G). Taken together, these findings indicate that MYPT1‐L promotes the proliferation and survival of HCC cells, whereas MYPT1‐S does not exhibit these oncogenic properties.

Given that RBM25 knockdown reduced the protein level of MYPT1‐L and its oncogenic function, we conducted rescue experiments to examine whether the phenotype resulting from RBM25 depletion could be rescued by MYPT1‐L overexpression. As expected, the elevated expression of MYPT1‐L significantly counteracted the inhibition of proliferation and clonogenicity ability caused by stable RBM25 knockdown in Huh7 cells (Figure 4H‐J). Similar results were obtained in Huh7 cells with inducible RBM25 knockdown (Figure S3H,I). Moreover, we explored the functional significance of MYPT1‐L in facilitating the oncogenic effects of RBM25 in vivo. We found that overexpressing MYPT1‐L rescued the xenograft growth inhibition caused by RBM25 knockdown (Figure 4K‐M, Figure S3J). We subsequently analyzed the splicing of MYPT1 and the clinical correlation with RBM25 and MYPT1‐L. When compared to paired adjacent normal tissues, we observed a shift in the splicing of MYPT1 in tumor samples, with MYPT1‐L emerging as the predominant isoform (Figure 4N). A positive correlation was found between the level of RBM25 protein and PSI of MYPT1 in HCC (Figure 4O). In addition, we analyzed TCGA data and confirmed that RBM25 expression was positively correlated with MYPT1‐L level (Figure S3K). Furthermore, higher levels of MYPT1‐L were closely associated with worse overall survival rates in patients with HCC (Figure 4P). Collectively, these findings suggest that RBM25 ablation inhibits HCC progression partially through switching the MYPT1 splicing pattern and reducing the expression of the MYPT1‐L isoform.

### 2.6 RBM25 Promotes MYPT1 Exon 13 Inclusion via direct Binding to its pre‐mRNA

**FIGURE 5:**
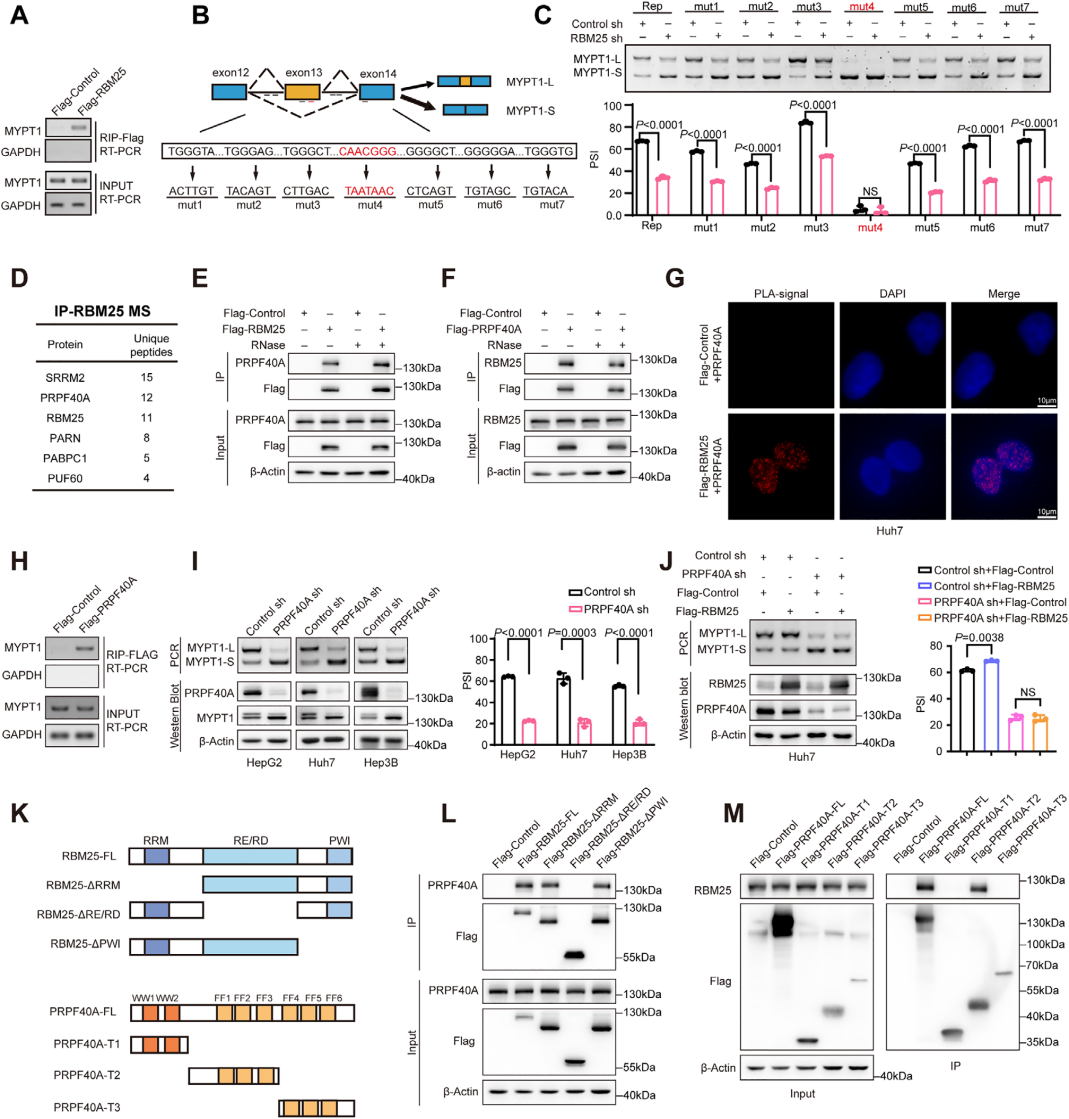
RBM25 interacts with PRPF40A to promote MYPT1 exon 13 inclusion. (A) Binding of MYPT1 pre‐mRNA with RBM25 was detected by RNA‐immunoprecipitation assay in cells exogenously expression FLAG‐RBM25 or vector control. (B) The schematic diagram shows the design of the MYPT1 splicing reporter. MYPT1 splicing reporter with the indicated mutations (mut 1–7) were generated. (C) MYPT1 splicing reporters containing various mutations were transfected in Huh7 cells with RBM25 knockdown to assay for the splicing change of MYPT1. The mean ± SD of PSIs from three experiments were plotted. A representative gel from triplicate experiments was shown. (D) List of RBM25 interactomes identified by immunoprecipitation‐coupled mass spectrometry (IP‐MS) using anti‐RBM25 antibody. (E) Immunoprecipitation was performed in 293T cells expressing Flag‐RBM25. (F) Immunoprecipitation was performed in 293T cells expressing Flag‐PRPF40A. (G) Proximity ligation assay (PLA) was performed to examine the interaction between Flag‐RBM25 and PRPF40A in Huh7 cells. (H) Binding of MYPT1 pre‐mRNA with PRPF40A was detected by RNA‐immunoprecipitation assay in cells exogenously expression Flag‐PRPF40A or vector control. (I) The splicing change of MYPT1 was detected by semi‐quantitative RT‐PCR and western blotting in HepG2, Huh7 and Hep3B cells with stable depletion of PRPF40A or control cells. The mean ± SD of PSIs from three experiments were plotted. (J) The splicing change of MYPT1 was examined in Huh7 cells expressing RBM25 with or without PRPF40A depletion (n = 3, mean ± SD). (K) The schematic illustrates the constructs of full‐length RBM25 and its truncations (ΔRRM, ΔRE/RD, ΔPWI), as well as full‐length PRPF40A and its truncation variants (T1‐T3). (L) Immunoprecipitation was performed in 293T cells expressing Flag‐RBM25 full length or truncations (ΔRRM, ΔRE/RD, ΔPWI) and the precipitated were analyzed.  (M) Immunoprecipitation was performed in 293T cells expressing Flag‐PRPF40A full length or truncations (T1‐T3) and the precipitated were analyzed. *P* values were determined by two‐tailed Student's t test (C, I), 1‐way ANOVA with Tukey's multiple comparison test (J).

To investigate the mechanisms of RBM25‐regulated splicing of MYPT1 gene, we performed an RNA‐immunoprecipitation assay and found that RBM25 indeed directly binds to the pre‐mRNA of endogenous MYPT1 (Figure 5A). Next, we constructed a minigene reporter spanning the genomic DNA fragment of MYPT1 exons 12–14 (Figure 5B). We then examined splicing following the transient transfection of HepG2 cells with either RBM25 knockdown or control. In line with the endogenous splicing pattern, the PSI of exon 13 was nearly 70% in control cells (Figure 5C, lane 1), whereas RBM25 knockdown indeed led to skipping of exon 13 (Figure 5C, lane 2). Data from ENCODE revealed that the RBM25 binding motif was poly G, as determined through an RNA Bind‐n‐Seq (RBNS) pulldown experiment against RBM25. Subsequent sequence analysis uncovered several potential RBM25‐binding motifs in MYPT1 pre‐mRNA (Figure 5B). To scrutinize the role of these internal binding motifs in exon inclusion, we designed a series of motif mutants of the MYPT1 minigene (mut 1‐mut 7) (Figure 5B). Remarkably, a mutant splicing reporter that disrupted the putative RBM25‐binding site (mut 4, CAACGGG to TAATAAC) abolished the splicing regulation of MYPT1 by RBM25, similar to the effect of RBM25 deprivation (Figure 5C). Collectively, our findings suggest that RBM25 regulates MYPT1 splicing through its binding to MYPT1 pre‐mRNA.

### 2.7 RBM25 Interacts With PRPF40A to Promote MYPT1 Exon 13 Inclusion

To better understand how RBM25 regulates the splicing of MYPT1, we pulled down RBM25‐containing immunocomplexes and determined its associated proteins using IP‐mass spectrometry assay (IP‐MS). In total, 27 specific RBM25‐interacting proteins were identified, with the top six proteins shown in Figure 5D. We identified PRPF40A as a potential interacting protein based on its capacity to bind proteins at the 5' and 3' splice sites, thereby promoting spliceosome assembly by bridging splice site recognition [20, 21]. The interaction between RBM25 and PRPF40A was confirmed by co‐IP assay (Figure 5E,F). Consistently, RBM25 was found to associate with endogenous PRPF40A in the nucleus in a PLA assay (Figure 5G and Figure S4A). While immunofluorescence (IF) analysis showed that RBM25 and PRPF40A were primarily localized in nuclear speckles, where they exhibited co‐localization (Figure S4B). RNA‐immunoprecipitation assay showed that PRPF40A could bind to the pre‐mRNA of MYPT1 (Figure 5H and Figure S4C). Furthermore, PRPF40A depletion led to MYPT1 exon 13 skipping in HepG2, Huh7, and Hep3B cells as judged by RT‐PCR (Figure 5I). PRPF40A reintroduction promoted MYPT1 exon 13 inclusion in PRPF40A‐depleted HepG2, Huh7 and Hep3B cells (Figure S4D). The expression levels of RBM25 and PRPF40A were unaffected by each other (Figure S4E,F). Collectively, these data indicate that RBM25 promotes MYPT1 exon 13 inclusion by interacting with PRPF40A.

To examine the functional significance of the interaction between RBM25 and PRPF40A, we assessed the effects of PRPF40A knockdown on RBM25‐mediated MYPT1 exon 13 regulation. In control cells transfected with empty pLKO.1 vector, overexpression of RBM25 promoted the inclusion of exon 13 in MYPT1 transcripts (Figure 5J). However, upon PRPF40A depletion, the ability of RBM25 to modulate MYPT1 exon 13 splicing was abolished (Figure 5J). These results indicate that the regulatory activity of RBM25 on MYPT1 exon 13 splicing is dependent on PRPF40A. To map the regions responsible for the RBM25‐PRPF40A interaction, we generated various truncated vectors of both proteins and found that the binding depends on the RE/RD domain of RBM25 and the first three FF domains of PRPF40A (Figure 5K‐M).

### 2.8 RBM25 Knockdown Suppresses YAP Signaling Through MYPT1 Splicing Switch

**FIGURE 6:**
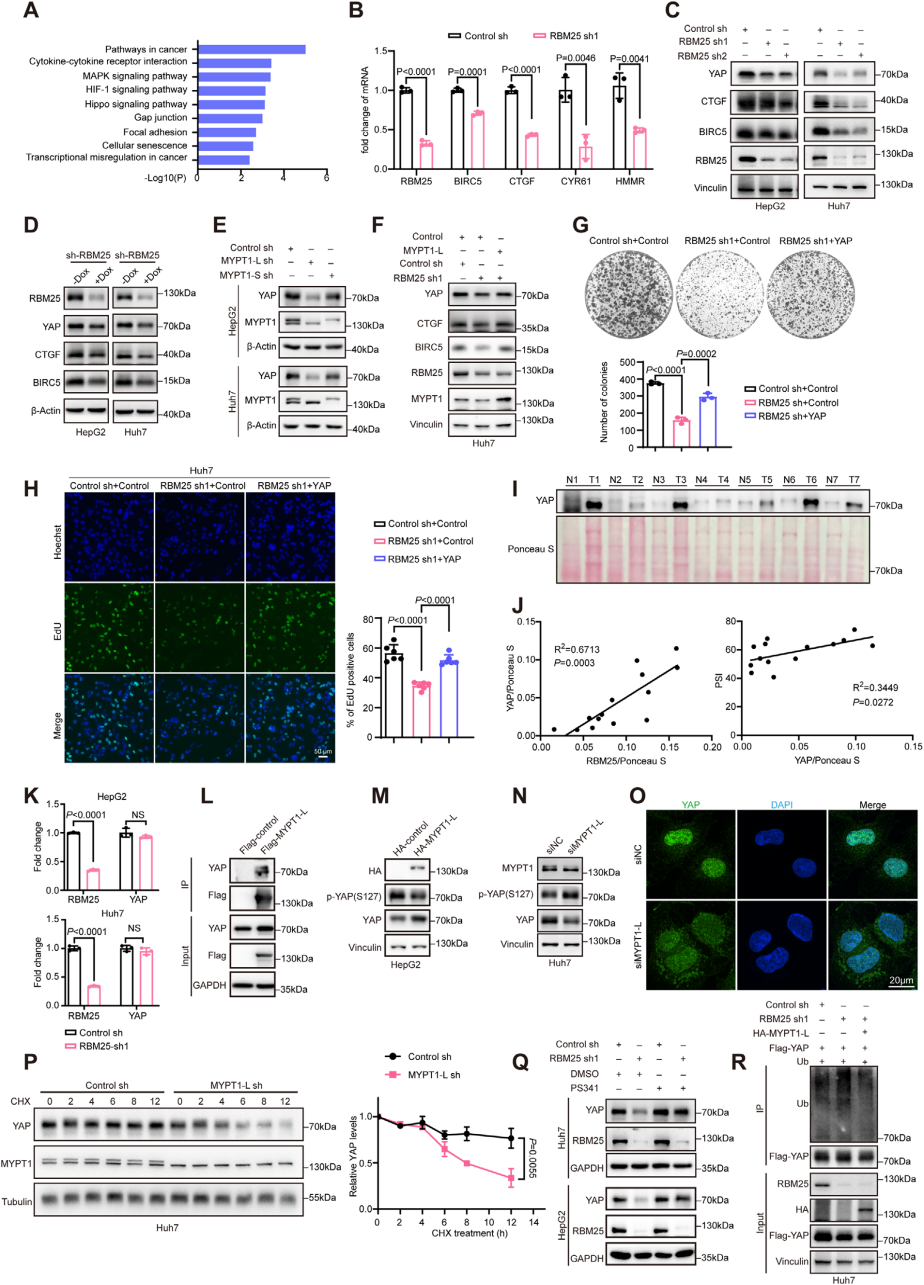
RBM25 knockdown inhibits YAP signaling through MYPT1 splicing switch. (A) KEGG pathway analysis of genes affected by RBM25. (B) Validation of various YAP targets in Huh7 cells with RBM25 knockdown by RT‐qPCR (n = 3, mean ± SD). (C) The levels of YAP and various YAP targets were examined in Huh7 and HepG2 cells with RBM25 knockdown by a western blot assay. (D) The levels of YAP and various YAP targets in Huh7 with DOX‐inducible depletion of RBM25 were measured with or without DOX treatment by a western blot assay. (E) The levels of YAP and various YAP targets were examined in Huh7 cells with MYPT1‐L or MYPT1‐S knockdown by a western blot assay. (F) The levels of YAP and various YAP targets were examined in RBM25‐depleted Huh7 cells with re‐expression of MYPT1‐L. (G) Colony formation assays using RBM25‐depleted Huh7 cells with or without YAP re‐expression were performed. Three experiments were carried out, with mean ± SD of colony numbers plotted. (H) The proliferative abilities of RBM25‐depleted Huh7 cells with or without YAP re‐expression were detected with an EdU staining assay. Three experiments were carried out, with mean ± SD of colony numbers plotted. (I) Protein levels of YAP in the paired fresh‐frozen HCC tissues (T) and the adjacent normal tissue specimens (N) from HCC patients were examined by a western blot assay. (J) Correlation between RBM25 levels in Figure 1C, PSI values of MYPT1 in Figure 4N and YAP protein levels was analyzed. (K) The mRNA levels of YAP and RBM25 were measured in Huh7 cells with RBM25 knockdown (n = 3, mean ± SD). (L) Co‐IP assay verifying the interaction between MYPT1‐L and YAP. (M, N) Western blot analysis of phosphorylated YAP upon MYPT1‐L overexpression (M) and MYPT1‐L knockdown (N). (O) Immunofluorescence assay showing YAP subcellular localization after MYPT1‐L depletion in Huh7 cells. (P) MYPT1‐L stably depleted Huh7 cells were treated with 100 µg/mL cycloheximide (CHX) at the indicated time points to detect the stability of YAP protein. YAP and MYPT1 levels were measured by immunoblotting. The intensity of YAP was quantified and plotted. Three experiments were conducted with mean ± SEM presented. (Q) The protein levels of YAP were measured in RBM25‐depleted Huh7 and HepG2 cells with or without the treatment of PS‐341. (R) Flag‐YAP or Flag‐YAP and MYPT1‐L were transiently co‐transfected with ubiquitin into RBM25 stably depleted Huh7 cells in the presence of PS‐341. Then Flag‐YAP was immunoprecipitated by anti‐FLAG M2‐beads followed by immunoblot using antibody against ubiquitin. *P* values were determined by two‐tailed Student's t test (B, K), 1‐way ANOVA with Tukey's multiple comparison test (G‐H), Pearson correlation test (J) and 2‐way ANOVA (P).

To better understand the intracellular signaling networks influenced by RBM25 in HCC progression, we analyzed the genes regulated by RBM25 using RNA sequencing data. We identified 736 genes with significant expression changes (|log_2_FoldChange| >1.5 with Padj < 0.05). KEGG analysis of the signaling pathways affected by RBM25 revealed that these genes were mostly enriched in several pathways including pathways in cancer, cytokine‐cytokine receptor interaction, MAPK signaling pathway, HIF‐1 signaling pathway, Hippo signaling pathway, gap junction, focal adhesion, cellular senescence and transcriptional misregulation in cancers (Figure 6A). The Hippo signaling pathway drew our attention due to its crucial role in cell growth, fate decision, organ size control, and regeneration [22, 23, 24, 25]. YAP is a key effector of the Hippo signaling pathway [17, 26]. Indeed, several YAP targets, such as CYR61, CTGF, BIRC5 and HMMR, were significantly downregulated by RBM25 knockdown as judged by RT‐qPCR (Figure 6B). Moreover, RBM25 depletion led to a reduction in YAP and its targets proteins in HepG2 and Huh7 cells (Figure 6C). A similar decrease in YAP and its target proteins was observed in HepG2 and Huh7 cells with inducible RBM25 knockdown (Figure 6D). Given the role of RBM25 in regulating MYPT1 splicing, we examined the protein levels of YAP following the depletion of MYPT1‐L and MYPT1‐S. We found that the specific knockdown of MYPT1‐L resulted in a reduction in the expression of YAP and its downstream targets, while MYPT1‐S knockdown had no impact on YAP expression (Figure 6E). Interestingly, we found that the decrease in YAP following RBM25 knockdown could be reversed by overexpressing MYPT1‐L (Figure 6F). We subsequently overexpressed YAP in cells with RBM25 depletion to determine whether the effect of RBM25 on HCC cell tumorigenesis was mediated by YAP. Remarkably, the overexpression of YAP significantly restored the proliferation capability of HCC cells with RBM25 knockdown (Figure 6G,H). In addition, we examined the YAP expression in human HCC samples (Figure 6I) and analyzed the correlation of RBM25, MYPT1 splicing, and YAP protein levels. Our results showed that RBM25 protein expression and the PSI of MYPT1 splicing were positively correlated with YAP protein abundance (Figure 6J). Furthermore, we analyzed TCGA data and confirmed that RBM25 and MYPT1‐L showed a positive correlation with the signature of YAP target genes (Figure S5A,B). Taken together, these findings suggest that RBM25 ablation inhibits HCC progression, at least in part, by suppressing YAP expression.

Given that depleted RBM25 significantly reduced YAP levels, we sought to investigate the molecular mechanism by which RBM25 regulates YAP expression. We first examined the mRNA level of YAP in HCC cells with RBM25 depletion. Interestingly, the mRNA level of YAP remained unaffected by RBM25 reduction in both HepG2 and Huh7 HCC cells (Figure 6K). Next, we investigated whether RBM25 influenced YAP protein levels through MYPT1‐L. We have performed additional experiments to establish the molecular link between RBM25‐MYPT1 splicing and YAP. Co‐IP assays verified the interaction between MYPT1‐L and YAP (Figure 6L). Western blot results showed that overexpression of MYPT1‐L reduced YAP phosphorylation, whereas MYPT1‐L knockdown increased YAP phosphorylation levels (Figure 6M,N). Immunofluorescence (IF) assays further demonstrated that MYPT1‐L depletion promoted YAP cytoplasmic translocation (Figure 6O). Collectively, these results suggest that MYPT1‐L modulates YAP phosphorylation status and its subsequent subcellular localization, thereby regulating YAP stability. We examined the effect of MYPT1‐L on the protein stability of YAP using a cycloheximide (CHX) chase assay. The turnover rate of the YAP protein was significantly faster in Huh7 cells with MYPT1‐L knockdown as compared to control cells (Figure 6P). We further examined whether RBM25‐mediated degradation of YAP is through the ubiquitin pathway and found that the downregulation of YAP induced by RBM25 depletion could be prevented by the treatment with PS‐341 (a proteasome inhibitor) (Figure 6Q). Furthermore, the increased ubiquitination of YAP triggered by RBM25 knockdown was effectively reversed through the overexpression of MYPT1‐L (Figure 6R). Taken together, our data suggest that knockdown of RBM25 switches the splicing of MYPT1 toward MYPT1‐S, thereby enhancing YAP protein degradation through the proteasome pathway.

### 2.9 Candicidin is an Inhibitor of RBM25 That Switches the Splicing of MYPT1 to Suppress HCC Cells Growth

**FIGURE 7:**
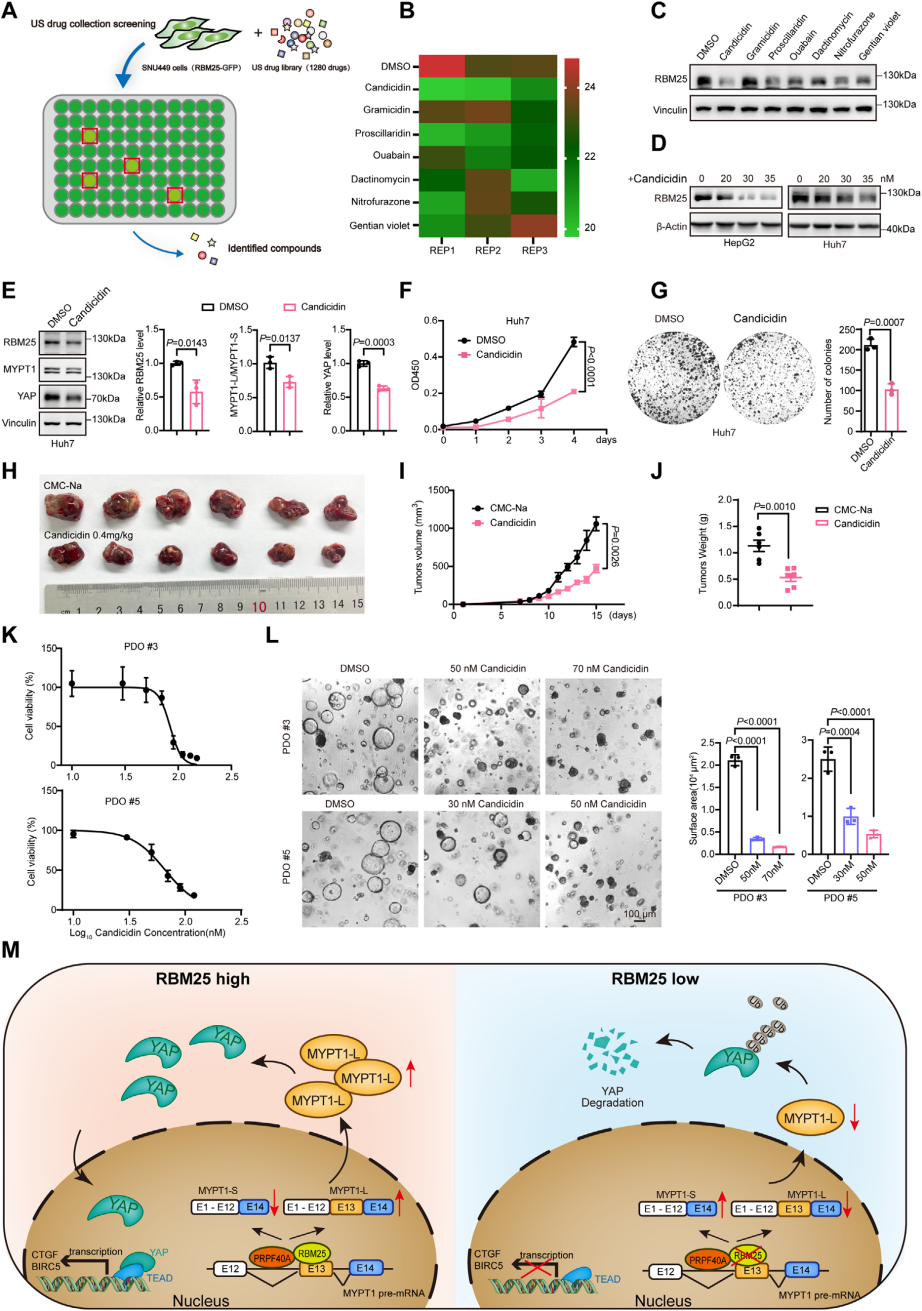
Candicidin is an inhibitor of RBM25 and inhibits HCC cells growth. (A) SNU449 cells expressing pEGFP‐RBM25 were treated with 1280 compounds and fluorescence intensities were measured. (B) Heatmap showing the GFP intensity in SNU449 cells treated with identified 7 compounds. (C) The protein levels of RBM25 in SNU449 cells with identified 7 compounds treatment were examined by a western blot assay. (D) HepG2 and Huh7 cells were treated with the indicated concentrations of candicidin, and RBM25 protein levels were detected. (E) The protein levels of RBM25, MYPT1 and YAP in Huh7 cells with 30 nM candicidin treatment were measured by a western blot assay. (F) The effect of candicidin on cell viability of Huh7 cells was detected by CCK‐8 (n = 5, mean ± SD). (G) Colony formation assays using Huh7 cells with or without candicidin treatment were performed. Three experiments were carried out, with mean ± SD of colony numbers plotted. (H‐J) Huh7 cells were subcutaneously injected into the flanks of NYG mice. When the tumors reached the volume of approximately 50 mm^3^, the mice were randomly and blindly divided into two groups (six per treatment group) and 100 µL 0.4 mg/kg candicidin in 0.5% CMC‐Na or 100 µL 0.5% CMC‐Na were administrated by oral gavage every day, respectively. (H) Pictures of removed tumors. (I) The average sizes of xenograft tumors were measured (n = 6, mean ± SEM). (J) Tumors were weighed and plotted (n = 6, mean ± SEM). (K) Dose response curve of HCC organoids treated with candicidin. (L) Representative images and size of HCC organoids treated with candicidin at indicated concentrations. (M) The schematic of how RBM25 regulates MYPT1 alternative splicing and YAP signaling in HCC. *P* values were determined by 2‐way ANOVA (F, I), two‐tailed Student's t test (E, G, J), and 1‐way ANOVA with Dunnett's multiple comparison test (L).

Considering the significant role of RBM25‐mediated MYPT1 splicing to stabilize YAP in suppressing HCC growth, we hypothesized that targeting RBM25 could be a promising strategy for HCC treatment. To test this, we performed a chemical screen to systematically identify potential inhibitors of RBM25 using the US drug collection of compounds (https://www.selleckchem.com/screening/fda‐approved‐drug‐library.html), which contains 1280 drugs [27]. We generated SNU449 HCC cells stably expressing EGFP‐RBM25 fusion protein (Figure S6A), and treated these cells with distinct compounds (each 1 µM concentration) for 12 h (Figure 7A). The top seven hits, namely candicidin, gramicidin, proscillaridin, ouabain, dactinomycin, nitrofurazone, and gentian violet were identified as they reduced the fluorescence of EGFP‐RBM25 (Figure 7B). Western blot analysis further confirmed that these seven compounds could decrease the protein level of RBM25 (Figure 7C).

Among these, candicidin, a polyene antifungal antibiotic produced by a strain of Streptomyces griseus, was identified as the most effective compound in suppressing the level of RBM25. The half maximal inhibitory concentration (IC50) of candicidin was found to be 55.32 nM in HepG2 cells and 33.04 nM in Huh7 cells (Figure S6B,C). We confirmed that candicidin reduced the level of endogenous RBM25 (Figure S6D). Furthermore, candicidin significantly downregulated the expression levels of RBM25 in a dose‐dependent manner (Figure 7D). As expected, candicidin also switched the splicing of MYPT1 and decreased YAP protein expression (Figure 7E and Figure S6E). Molecular docking analysis revealed that candicidin could directly bind to RBM25 protein with high affinity (Figure S6F). Treatment with the proteasome inhibitor MG132 restored RBM25 protein levels (Figure S6G), indicating that candicidin downregulates RBM25 expression through promoting proteasome‐dependent degradation. Moreover, we examine the expression of several splicing factors including RBM39, hnRNPU, SRSF4 and SRSF1, and found that candicidin did not obviously affect their protein abundance (Figure S6H), indicating that candicidin specifically reduces the level of RBM25 rather than exerting a non‐specific global downregulation of splicing factors. Importantly, candicidin inhibited the growth of HepG2 and Huh7 cells as judged by CCK‐8 and colony formation assays (Figure 7F,G and Figure S6I,J). In line with the in vitro findings, candicidin significantly inhibited cell proliferation and tumor growth in Huh7 xenograft mouse model, as indicated by decreased tumor growth rates and smaller tumor masses at end point analysis (Figure 7H‐J). We also monitored the body weight of the mice and found that candicidin had no effect on it (Figure S6K). To further evaluate the anticancer effects of candicidin, we examined the expression of RBM25 via western blot (Figure S6L) and tested its impact on tumor organoids derived from two HCC patients. Importantly, candicidin treatment resulted in a dose‐dependent decrease in cell viability (Figure 7K), and a marked reduction in organoid size (Figure 7L). Altogether, our data suggest that the inhibition of RBM25 by candicidin suppressed tumor growth, thereby providing a potentially novel and effective therapeutic agent for HCC.

## 3 Discussion

Deregulated RNA splicing is a molecular characteristic that contributes to every aspect of cancer progression, and targeting splicing has emerged as a novel approach for cancer therapy [5, 6]. Accumulating evidence has indicated that deregulated alternative splicing profiles contribute to tumor initiation and progression, significantly increasing the complexity of oncogenic networks [5, 28]. Deciphering key oncogenic network facilitates the development of specific inhibitors targeting splicing. In this study, we conducted a comprehensive study of the splicing factor RBM25 and its downstream AS landscape in HCC. We identified a key AS target, MYPT1, whose full‐length isoform (MYPT1‐L) was closely associated with HCC progression and poor prognosis of patients. We concluded that RBM25 promotes hepatocarcinogenesis by controlling AS of tumor‐related genes, further substantiating the crucial role of AS in tumorigenesis.

AS is generally regulated by splicing factors that specifically bind to *cis*‐elements in pre‐mRNA, thereby affecting the selection of adjacent splice sites [29, 30, 31]. Abnormal expression or activity of these splicing factors often leads to deregulated splicing, thereby promoting oncogenic transformation [32]. For example, the splicing factor SRSF1 is upregulated in various human tumors and plays oncogenic roles by controlling the splicing of several cancer‐related genes including BIN1, MKNK2, S6K1 and MYO1B [33, 34]. Whereas the splicing factor RBM4 is downregulated in multiple tumors and functions as a tumor suppressor by controlling AS events that are critical for cell proliferation, migration and apoptosis [35, 36]. In this study, we identified RBM25 as an oncogenic splicing factor in HCC progression. RBM25 is significantly upregulated in HCC, and its overexpression is associated with poor survival of HCC patients. The subsequent functional study confirmed that RBM25 knockdown inhibits HCC cell proliferation in vitro and suppresses the growth of diethylnitrosamine and carbon tetrachloride (DEN/CCL_4_) induced HCC models in vivo. Additionally, overexpression of RBM25 promoted the proliferation of HCC cells. These findings indicate that RBM25 acts as a proto‐oncogene, playing a crucial role in HCC progression.

Considering the context‐dependent of RBM25 target selection, the key RBM25‐regulated AS networks responsible for tumorigenesis vary greatly across different tumor types. Although several AS targets of RBM25, including Bcl‐x and BIN1 [12, 14], have been identified, the effect of RBM25 on AS in HCC remains largely unknown. In this study, we conducted a comprehensive analysis of the RBM25‐regulated AS events in HCC cells by integrating the RNA‐seq data and validation methods. Most of these events belong to the skipped exon category, and there was a decrease in the inclusion of alternatively spliced exons following RBM25 knockdown. Such finding is consistent with previous report [13], suggesting that RBM25 functions as a splicing activator. Among the numerous AS targets regulated by RBM25 in HCC, we focus on the *MYPT1* gene, as its mature mRNA products dramatically switched to the exon‐skipped isoform upon RBM25 depletion. MYPT1 serves as an essential regulatory subunit of the myosin light chain phosphatase (MLCP) protein complex and plays a role in regulating smooth muscle relaxation [19]. Recent studies have revealed additional roles of MYPT1, including its involvement in migration and cell adhesion [37], cell cycle regulation [38], and anoikis resistance [39]. As the core regulatory subunit of MLCP, MYPT1 dictates the substrate specificity, subcellular localization, and activity of phosphatase complex [19]. Exon 13 skipping reshapes the serine/threonine‐rich domain of MYPT1, altering its intrinsic phosphorylation status, protein interaction capacity and downstream substrate recognition. This splicing switch broadly perturbs multiple intracellular signaling cascades, thereby exerting influences on malignant biological behaviors of HCC cells.

In our study, we found that upregulation of RBM25 promotes the inclusion of exon 13 in MYPT1 by interacting with PRPF40A, thereby leading to increased expression of the long isoform of MYPT1 (MYPT1‐L), which promotes HCC progression (Figure 7M). PRPF40A is a component of spliceosomal complexes, and the interaction between RBM25 and PRPF40A may effectively stabilize spliceosome assembly near the splice site of MYPT1 exon 13, thereby promoting exon inclusion. Loss of this interaction upon RBM25 deficiency thus shifts the balance toward exon skipping, leading to preferential production of MYPT1‐S.

MYPT1 is dynamically modified by O‐GlcNAc, which inhibits MYPT1 phosphorylation and maintains its activity [40]. Exon 13, which encodes the serine/threonine region, contains eight identified O‐GlcNAc sites that inhibit MYPT1 phosphorylation and maintain its activity. More importantly, our study identifies the Hippo pathway effector YAP as a critical downstream target of RBM25‐MYPT1 splicing axis. MYPT1‐L interacts with YAP and promotes its dephosphorylation and stabilization, thereby facilitating YAP nuclear translocation and activating downstream oncogenic transcriptional programs to drive HCC development. Collectively, these results establish a link between RBM25‐mediated splicing of MYPT1 and Hippo pathway activation in HCC.

In addition to the events regulated by RBM25, other events may also play key roles in HCC progression. Our gene ontology analysis revealed that RBM25 influences numerous AS events related to the cell cycle and histone modification. For example, KDM6A, an H3K27 histone demethylase [41, 42], also affects H3K4me1 methyltransferase activity by interacting with KMT2D in the COMPASS complex [43, 44]. KDM6A is a crucial tumor suppressor frequently mutated in various human cancers, including HCC, gastrointestinal cancers, and small cell lung cancer [44, 45, 46]. Knockdown of RBM25 promotes the production of full‐length isoform of KDM6A, a tumor suppressor. As a master regulator of cancer‐related AS, RBM25 potentially promotes tumorigenesis through multiple oncogenic pathways, which helps to explain why co‐expression of MYPT1‐L only partially reversed the phenotype.

In summary, our study demonstrated that the splicing factor RBM25 is usually upregulated in HCC patients and its overexpression is associated with poor prognosis. RBM25 promotes HCC progression by facilitating the inclusion of exon 13 in MYPT1 pre‐mRNA, leading to the generation of the oncogenic MYPT1‐L isoform, which stabilizes YAP (Figure 7M). Our findings suggest that RBM25 is a critical oncogenic splicing factor that could serve as a prognostic biomarker and potential therapeutic target in HCC.

## 4 Experimental Section

### 4.1 Cell Culture and Construction of Stable Cell Lines

HCC cell lines HepG2 (CVCL_0027), Huh7 (CVCL_0336), Hep3B (CVCL_0326) and HEK293T (CVCL_0063) cells were from National Collection of Authenticated Cell Cultures, Chinese Academy of Sciences. SNU449 (CVCL_0454) cells were purchased from the American Type Culture Collection (ATCC). HCCLM9 (CVCL_A5CU) cells [47, 48, 49] were kindly provided by Prof. Shimei Zhuang from Sun Yat‐sen University and Prof. Yong Zeng from the Laboratory of Liver Surgery, West China Hospital, Sichuan University. HepG2 and Hep3B cells were maintained in MEM medium supplemented with 1 mM sodium pyruvate, 1% nonessential amino acids and 10% fetal bovine serum (FBS, Gibco), and Huh7 cells and HEK293T were cultured in DMEM (Gibico) medium with 10% FBS under standard culture conditions (37°C, 5% CO_2_). To construct RBM25 or MYPT1‐L stable‐knockdown cell lines, shRNAs targeting RBM25, MYPT1‐L or MYPT1‐S were cloned into the pLKO.1 to gain pLKO.1‐RBM25, pLKO.1‐MYPT1‐L or MYPT1‐S vectors. The shRNA targeting MYPT1‐L was designed to bind the sequence of exon 13, while the shRNA targeting MYPT1‐S was designed to recognize the unique splice junction generated by exon 13 skipping. Primers are listed in Table S1. HEK293T cells were transfected with pLKO.1‐RBM25 or pLKO.1‐MYPT1‐L constructs, together with pPAX2 and pMD2 lentiviral packaging systems. Viruses with shRNA for RBM25 or MYPT1‐L were harvested after 72 h transfection and used to infect Huh7 and Hep3B cells, followed by 4 µg/mL puromycin (Solarbio, P8230) selection for 5 days. To generate the doxycycline (DOX)‐inducible RBM25‐knockdown stable cell lines, shRNAs targeting RBM25 or MYPT1‐L were cloned into the pLKO‐Tet‐On to gain pLKO‐Tet‐On‐RBM25‐shRNA vectors. Then HEK293T cells were transfected with pLKO‐Tet‐On‐RBM25‐shRNA vectors for 72 h. The supernatant containing virus was collected and used to infect Huh7 cells. The stably integrated cells were selected with 4 µg/mL puromycin (Solarbio, P8230) for 5 days and then cultured in medium containing 2 µg/mL puromycin in a 5% CO_2_ incubator at 37°C.

### 4.2 Cell Proliferation, EdU Assays and Colony Formation Assays

For cell growth, Hep3B or Huh7 cells (5 × 10^3^ cells/well) were plated in 24‐well plates for incubation at 37°C and counted every two days to determine the cell growth curve. For CCK‐8 assays, HepG2 or Huh7 cells (1 × 10^3^ cells/well) were seeded in 96‐well plates. Cells were treated with CCK‐8 reagent (10 µL/well) at 37°C for 2 h and detected at wavelengths of 450 nm with an enzyme‐labeled instrument, which was measured every day for 6 days. For EdU assays, HepG2 or Huh7 cells (5 × 10^3^ cells/well) were seeded in 96‐well plates and incubated for 24 h. Cells were treated with 50 µM EdU reagent for 2 h, fixed with 4% PFA and stained with 1 × Apollo dye solution and 1 × Hoechst 33342 solution according to the manufacturer's protocol (RiboBio). Finally, Fluorescence photographs were taken with a Leica microscope, and the positivity rate was calculated by manual quantification of positive cells with fluorescence using ImageJ. For the colony formation assay, cells (3 × 10^3^ cells/well) were seeded into 6 cm plates and incubated for more than one or two weeks. Colonies were fixed with 4% PFA, stained with 0.1% crystal violet and washed with running water. Finally, colonies were photographed to make statistics for cell colonies.

### 4.3 Western Blot

Cells were lysed with RIPA lysis buffer containing 1 mM Na_3_VO_4_, 1 mM Cocktail, and 1 mM PMSF for 20–30 min and centrifuged at 12 000 g for 20 min to remove cell debris. The protein samples (30–50 µg) were fractionated by 8% or 10% SDS‐PAGE after boiling for 5 min in 1 × SDS sample buffer and transferred to NC membranes. NC membranes with protein samples were incubated with primary antibodies at 4°C overnight after being blocked with 5% milk in PBST. The primary antibodies used were anti‐RBM25 (1:1000, Proteintech, 25297‐1‐AP), anti‐MYPT1 (1:1000, ABclonal, A0587), anti‐YAP(1:1000, CST, 4912S), anti‐CTGF(1:1000, HUABIO, ER1802‐69), anti‐BIRC5(1:1000, CST, 2808S), anti‐p‐YAP(S127) (1:1000, CST, 4911S), anti‐PRPF40A(1:3000, Proteintech, 17392‐1‐AP), anti‐Vinculin (1:5000, Proteintech, 66305‐1‐Ig), anti‐Tubulin(1:1000, ABclonal, AC006), anti‐β‐Actin (1:10000, Proteintech, 20536‐1‐AP), anti‐Flag (1:1000, Sigma‐Aldrich, F1804), anti‐HA(1:1000, Covance, mms‐101p‐1). The membranes were washed with PBST 3 times for 5 min each and probed with secondary antibodies coupled to horseradish peroxidase (HRP) for 1 h at room temperature. Finally, the membranes were washed with PBST 3 times for 5 min each, and specific protein bands were visualized with the ECL enhanced chemiluminescence reagent Kit (NCM Biotech) and MiniChemi Chemiluminescence imager (SageCreation, Beijing).

### 4.4 RT‐PCR

Total RNA was extracted from RBM25‐knockdown cells using Trizol reagent (Invitrogen) according to the manufacturer's instructions. Total RNA (2 µg) was reverse transcribed to get cDNA with PrimeScript RT reagent kit (Takara) after genome DNAs removed. Then cDNA as the template was measured by PCR amplification using 2 × EasyTaq PCR SuperMix (TRAN), and PCR products were separated on 2% gels. The amount of each splicing isoform was measured based on band intensities of PCR products using the ImageJ. Splicing outcomes were determined by estimating percent‐spliced‐in (PSI) values that were calculated by the ratio of the amount of long isoform to the amount of both long isoform and short isoform. Primers are listed in Table S1.

### 4.5 Xenograft Tumor Formation

The experimental protocol on animals was approved by the Institutional Animal Care and Use Committee of Dalian Medical University (approval no. AEE21015). 3 × 10^6^ DOX‐inducible RBM25‐knockdown stable Huh7 cells were injected subcutaneously into the left flank of 5‐week‐old male NYG mice. Mice were randomly assigned to experimental groups using a simple randomization procedure. To induce RBM25 knockdown, mice were fed with 0.1% DOX in 3% sucrose solution. Control groups received 3% sucrose solution without DOX. Tumor size was monitored by blinded caliper measurements of the subcutaneous mass. All mice were sacrificed after 15 days, and tumors were removed for further analysis. Tumor volume (V) was calculated using the equation V (mm^3^) = a × b^2^/2, where a and b are the largest diameter and smallest diameter. For the rescue experiment in vivo, DOX‐inducible RBM25‐knockdown stable Huh7 cells, either with MYPT1‐L overexpression or with pCDH vector, were subcutaneously injected into the left flank of 5‐week‐old NYG mice. On the subsequent day, mice bearing DOX‐inducible RBM25‐knockdown stable Huh7 cells carrying the pCDH vector were fed either 0.1% DOX in 3% sucrose aqueous solution to induce RBM25 knockdown or 3% sucrose aqueous solution alone as the control. Mice bearing MYPT1‐L‐overexpressing RBM25‐knockdown Huh7 cells were fed with 0.1% DOX in 3% sucrose aqueous solution. The 0.1% DOX solution was changed every 2 days, and the size of the tumor was measured. All mice were sacrificed after 15 days, and tumors were collected and subjected to IHC and H&E staining.

### 4.6 Spontaneous HCC Mouse Models

The experimental protocol on animals was approved by the Institutional Animal Care and Use Committee of Dalian Medical University (approval no. AEE21015). CRISPR‐mediated Rbm25 knockout mice (*Rbm25^+/−^*) in the C57BL/6 strain background were obtained from Cyagen Biosciences. Fourteen‐day‐old male mice (*Rbm25^+/−^* and *Rbm25^+/+^*) were injected once with diethylnitrosamine (DEN, 20 mg/kg, dissolved in PBS), and 20% CCl_4_ in olive oil was intraperitoneally injected once a week for 16 weeks at the dosage of 10 µL/g when mice were 5 weeks old. The DEN combined CCl_4_ models were sacrificed when mice were 40 weeks old. After sacrifice, liver weight, body weight, maximum tumor nodule size as well as surface tumor nodule numbers were recorded, and livers were collected and subjected to IHC and H&E staining.

### 4.7 Immunohistochemical Staining

For tissue specimens, fresh tissues collected were fixed in 10% formaldehyde solution overnight and washed with running water for 30 min. Tissues were transferred into 70% ethanol, which was followed by paraffin embedment including dehydration, clearing, wax immersion and embedding. Paraffin‐embedded tissue specimens were sectioned for Immunohistochemical staining. For IHC staining, tissue sections or human liver cancer tissue array (HLiv‐HCC180Sur‐03) were deparaffinized in xylene and rehydrated followed by antigen retrieval in sodium citrate. Next, sections were processed according to the manufacturer's instructions (One‐Step IHC Assay Kits, KeyGEN), in which the primary antibodies used were anti‐RBM25 (1:100, Proteintech, 25297‐1‐AP), anti‐Ki67 (1:1000, CST, #9449) and anti‐AFP (1:100, Proteintech, 14550‐1‐AP).

### 4.8 Proximity Ligation Assay (PLA)

Cells were cultured on sterile glass cover slips, and fixed with 4% paraformaldehyde for 10 min, permeabilized in 0.2% Triton X‐100 for 10 min at 4 °C. Then the cells were subsequently processed as per manufacturer's instructions (DUO92101 Sigma–Aldrich). Primary antibodies used as follows: anti‐RBM25 (Proteintech, 25297‐1‐AP) and PRPF40A (Proteintech, 17392‐1‐AP). Primary antibodies were diluted to 1:100. Cells were visualized and images were captured using Leica microscope (Leica DMi8, Germany).

### 4.9 Screening of US Drug Collection of Compounds Against RBM25

The human *RBM25* gene was subcloned into the pCDH‐GFP vector to establish the pCDH‐GFP‐RBM25 plasmid. HEK293T cells were transfected with pCDH‐GFP‐RBM25 plasmid, together with pPAX2 and pMD2 lentiviral packaging systems. Viruses were harvested after 72 h transfection and used to infect SNU449 cells, followed by 4 µg/mL puromycin (Solarbio, P8230) selection for 5 days, to construct SNU449 cell line with GFP‐RBM25 stably expressed. The stable SNU449 cells were plated in 96‐well plates. When cells grew well and were at 90% confluence, individual drugs were added to each well for 12 h (1 µM). Cells treated with drugs were washed with PBS, and RBM25 expression was determined by fluorescence intensity which was detected by Leica microscope (Leica Mi8) and semi‐quantitatively analyzed by ImageJ. Relative fluorescence intensity was normalized to that of DMSO‐treated cells, and all experiments were performed in triplicate and repeated three times.

### 4.10 Statistical Analysis

GraphPad Prism 10.4 was used to analyze the data presented as mean ± SD/SEM. The sample size (n) for each group is indicated in corresponding figure legends. Comparisons between 2 groups were determined by 2‐tailed unpaired t‐test or 2‐tailed paired t‐test. For comparisons among three or more groups, one‐way ANOVA followed by Dunnett's multiple comparison test (vs. single control group) or Tukey's multiple comparison test (all pairwise comparisons) was applied. Two‐way ANOVA was used to analyze the differences of multi‐groups with the change of time. *P* < 0.05 was regarded as statistically significant.

### 4.11 Ethics Approvals

The Institutional Animal Care and Use Committee of the Dalian Medical University approved the use of animal models in this study (approval no. AEE21015). All human tumor tissues were obtained with written informed consent from patients or their guardians prior to participation in the study. The Institutional Review Board of the First Affiliated Hospital of Dalian Medical University approved the use of the tumor specimens in this study (approval no. PJ‐KS‐KY‐2022‐208).

## Author Contributions

**Wenjing Zhang**: conceptualization, methodology, investigation, writing – review and editing, funding acquisition, writing – original draft, data curation. **Lili Zhi**: methodology, investigation, validation, data curation. **Tian Huang**: methodology, investigation, validation, data curation. **Lingya Feng**: methodology, investigation, validation, data curation. **Chaoqun Chen**: methodology, formal analysis, data curation. **Huanhuan Wei**: methodology, investigation, funding acquisition. **Xiaolong Liu**: methodology, investigation, funding acquisition. **Lei Chen**: methodology, data curation. **Jinrui Zhang**: investigation, methodology. **Ge Zhang**: methodology, investigation. **Baofeng Zhao**: data curation. **Rong Liu**: data curation. **Dan Chen**: conceptualization, methodology, data curation, writing – review and editing, funding acquisition. **Yangfan Qi**: conceptualization, methodology, investigation, data curation, funding acquisition, writing – review and editing. **Yang Wang**: conceptualization, methodology, supervision, formal analysis, funding acquisition, writing – original draft, writing – review and editing.

## Conflicts of Interest

The authors declare no conflicts of interest.

## Supporting information




**Supporting File 1**: advs77792‐sup‐0001‐SuppMat.pdf.

**Supporting File 2**: advs77792‐sup‐0002‐TableS1.xlsx.

## Data Availability

The data that support the findings of this study are openly available in Gene Expression Omnibus of NCBI at https://www.ncbi.nlm.nih.gov/geo/, reference number GSE284351.
