# Supplementary material for: RBM25 Drives Hepatocellular Carcinoma Progression by Stabilizing YAP Through Regulating Oncogenic Splicing‐switch of MYPT1
Source: Adv Sci (Weinh). 2026 Sep 16:e77792. Online ahead of print. doi: 10.1002/advs.77792 (PMC13583231; doi:10.1002/advs.77792)
Supplement: Supplementary file 1 [file ADVS-9999-e77792-s002.docx]

Supporting Information

RBM25 Drives Hepatocellular Carcinoma Progression by Stabilizing YAP through Regulating Oncogenic Splicing-switch of MYPT1

Wenjing Zhang*, Lili Zhi, Tian Huang, Lingya Feng, Chaoqun Chen, Huanhuan Wei, Xiaolong Liu, Lei Chen, Jinrui Zhang, Ge Zhang, Baofeng Zhao，Rong Liu, Dan Chen*, Yangfan Qi*, Yang Wang*

**Figure S1. Overexpression of RBM25 correlates with poor prognosis in patients with HCC.** (**A**) Kaplan-Meier analysis of the OS (overall survival, n = 364), RFS (relapse free survival, n = 316), DSS (disease-specific survival, n = 362) and PFS (progression free survival, n = 370) of HCC patients with high or low RBM25 expression based on RNA-seq data. (**B**) The schematic of generation of RBM25 knockout mice. (**C**) The validation of mice with RBM25 deletion by PCR and DNA sequencing. (**D**) Genotyping of RBM25 wild type (WT) and RBM25 heterozygous deletion (HE) mice. Three primers (F1, R1 and R2) were used for RBM25 genotyping, as shown in Supplementary Figure 1B.

**Figure S2. RBM25 ablation inhibits the growth of HCC cells.** (**A**) Cell viabilities of Hep3B and HCCLM9 cells with RBM25 knockdown were measured by the CCK-8 growth curve assays (n = 3, mean ± SD). (**B**) Colony formation assays using Hep3B and HCCLM9 cells with stable depletion of RBM25 were performed (n = 3, mean ± SD). (**C**) Cell proliferation of Hep3B and HepG2 cells with RBM25 overexpression was measured (n = 5, mean ± SD). (**D**) Colony formation assays using Hep3B and HepG2 cells with RBM25 overexpression were performed. Three experiments were carried out, with mean ± SD of colony numbers plotted. (**E**) Body weight variation of the mice during the treatment (mean ± SEM, n=6). *P* values were determined by 2-way ANOVA (**A**, **C**), 1-way ANOVA with Dunnett’s multiple comparison test (**B**), or two-tailed Student’s t test (**D**).

**Figure S3. Depletion of RBM25 inhibits liver cancer progression partially through MYPT1.** (**A**-**B**) Semi-quantitative RT-PCR was used to detect MYPT1 splicing patterns. Western blotting was used to measure RBM25 protein level in RBM25-depleted HepG2, Huh7 and Hep3B cells with RBM25 re-expression. The mean ± SD of PSIs from three experiments were plotted. (**C**) Cell proliferation of Hep3B and Huh7 cells with MYPT1-L or MYPT1-S overexpression was measured (n = 5, mean ± SD). (**D**) Colony formation assays using Hep3B and Huh7 cells with MYPT1-L or MYPT1-S overexpression were performed. Three experiments were carried out, with mean ± SD of colony numbers plotted. (**E**) Cell proliferation of HepG2 and Huh7 cells with MYPT1-L knockdown was measured by CCK-8 (n = 4, mean ± SD). (**F**) Colony formation assays using Hep3B and Huh7 cells with MYPT1-L knockdown were performed. Three experiments were carried out, with mean ± SD of colony numbers plotted. (**G**) Body weight changes in mice subcutaneously injected with Huh7 cells with expression of MYPT1-L or MYPT1-S (mean ± SEM, n=6). (**H**) The protein levels of RBM25, HA-MYPT1-L and Vinculin were examined by a western blot assay. (**I**) Colony formation assays using inducible RBM25-depleted Huh7 cells with or without MYPT1-L re-expression were performed (n = 3, mean ± SD). (**J**) Body weight changes in NYG mice subcutaneously injected with inducible RBM25-depleted Huh7 cells with or without MYPT1-L re-expression (mean ± SEM, n=5). (**K**) Correlation between RBM25 expression and MYPT1-L expression levels in 419 LIHC patients in TCGA. Three experiments were carried out, with mean ± SD of colony numbers plotted. *P* values were determined by 1-way ANOVA with Dunnett’s multiple comparison test (**B**, **D**), 2-way ANOVA (**C**, **E**) , two-tailed Student’s t test (**F**), and 1-way ANOVA with Tukey’s multiple comparison test (**I**).

**Figure S4. RBM25 promotes MYPT1 exon 13 inclusion by interacting with PRPF40A.** (**A**) Proximity ligation assay (PLA) was performed to examine the interaction between Flag-RBM25 and PRPF40A in HepG2 and Hep3B cells. Scale bar: 10 μm. (**B**) Immunofluorescence staining was used to determine the subcellular localization of RBM25 and PRPF40A. Scale bar: 10 μm. (**C**) Western blotting was used to determine the Flag-PRPF40A expression. (**D**) The splicing change of MYPT1 was detected in PRPF40A-depleted HepG2, Huh7 and Hep3B cells with PRPF40A re-expression. The mean ± SD of PSIs from three experiments were plotted. *P* values were determined by 1-way ANOVA with Dunnett’s multiple comparison test. (**E**) The protein levels of RBM25 and PRPF40A were measured in RBM25 stably depleted HepG2, Huh7 and Hep3B cells. (**F**) The protein levels of RBM25 and PRPF40A were measured in PRPF40A stably depleted HepG2, Huh7 and Hep3B cells.

**Figure S5. TCGA-based correlation analysis of RBM25, MYPT1-L and the YAP target gene signature.** (**A**) Scatter plot illustrating the Spearman’s correlation between RBM25 expression and YAP target gene signature ((MSigDB ID:M2871)) using the TCGA LIHC cohort dataset. (**B**) Scatter plot showing the correlation between MYPT1-L expression and the YAP targets gene signature (MSigDB ID:M2871) using the TCGA LIHC cohort dataset.

**Figure S6. Candicidin is an inhibitor of RBM25 and inhibits HCC cells growth.** (**A**) SNU449 cells expressing pEGFP-RBM25 were detected. (**B**) IC50 of candicidin in HepG2 cells were determined by CCK-8. (**C**) IC50 of candicidin in Huh7 cells were determined by CCK-8. (**D**) The endogenous RBM25 were detected in Huh7 cell with 20 nM and 40 nM candicidin by an immunofluorescence assay. (**E**) The protein levels of RBM25, MYPT1 and YAP in HepG2 cells with 35 nM candicidin treatment were measured by a western blot assay (n = 3, mean ± SD). (**F**) Molecular docking analysis between RBM25 and candicidin using AutoDock Vina (affinity: -8.4 Kcal/mol). (**G**) The protein levels of RBM25 were measured in Huh7 cells treated with 30 nM candicidin in the presence or absence of MG132. (**H**)Expression levels of splicing factors (RBM25, RBM39, hnRNPU, SRSF4 and SRSF1) in HepG2 cells after treatment with 35 nM. (**I**) The effect of candicidin on cell viability of HepG2 cells was detected by CCK-8 (n = 5, mean ± SD). (**J**) Colony formation assays using HepG2 and Huh7 cells with or without candicidin treatment were performed. Three experiments were carried out, with mean ± SD of colony numbers plotted. (**K**) The effect of candicidin on the weight of NYG mice (mean ± SEM, n=6). (**L**) The protein levels of RBM25 in PDOs were determined by a western blot assay.
